# Bacterial dynamics during the burial of starch-based bioplastic and oxo-low-density-polyethylene in compost soil

**DOI:** 10.1186/s12866-022-02729-1

**Published:** 2022-12-20

**Authors:** Joshua Abednego Wicaksono, Tresnawati Purwadaria, Adi Yulandi, Watumesa Agustina Tan

**Affiliations:** 1 Master of Biotechnology Program, Faculty of Biotechnology, Atma Jaya Catholic University of Indonesia, BSD Campus, Jalan Raya Cisauk – Lapan no. 10, Tangerang, Indonesia; 2 Biotechnology Program, Faculty of Biotechnology, Atma Jaya Catholic University of Indonesia, BSD Campus, Jalan Raya Cisauk – Lapan no. 10, Tangerang, Indonesia

**Keywords:** Starch-based bioplastic, Oxo-low-density polyethylene, Soil metagenome, Bacterial dynamics

## Abstract

**Background:**

Plastic waste accumulation is one of the main ecological concerns in the past decades. A new generation of plastics that are easier to degrade in the environment compared to conventional plastics, such as starch-based bioplastics and oxo-biodegradable plastics, is perceived as a solution to this issue. However, the fate of these materials in the environment are unclear, and less is known about how their presence affect the microorganisms that may play a role in their biodegradation. In this study, we monitored the dynamics of bacterial community in soil upon introduction of commercial carrier bags claimed as biodegradable: cassava starch-based bioplastic and oxo-low-density polyethylene (oxo-LDPE). Each type of plastic bag was buried separately in compost soil and incubated for 30, 60, 90, and 120 days. Following incubation, soil pH and temperature as well as the weight of remaining plastics were measured. Bacterial diversity in soil attached to the surface of remaining plastics was analyzed using Illumina high-throughput sequencing of the V3-V4 region of 16SrRNA gene.

**Results:**

After 120 days, the starch-based bioplastic weight has decreased by 74%, while the oxo-LDPE remained intact with only 3% weight reduction. The bacterial composition in soil fluctuated over time with or without the introduction of either type of plastic. While major bacterial phyla remained similar for all treatment in this study, different types of plastics led to different soil bacterial community structure. None of these bacteria were abundant continuously, but rather they emerged at specific time points. The introduction of plastics into soil increased not only the population of bacteria known for their ability to directly utilize plastic component for their growth, but also the abundance of those that may interact with direct degraders. Bacterial groups that are involved in nitrogen cycling also arose throughout burial.

**Conclusions:**

The introduction of starch-based bioplastic and oxo-LDPE led to contrasting shift in soil bacterial population overtime, which may determine their fate in the environment.

## Background

The widespread use of petroleum-based plastics has led to global pollution issues. In 2015, global plastic waste production is estimated at 25 billion metric tons, with only 9% of them recycled, 12% incinerated, and 79% ended up in landfills or natural environment [1]. Single-use plastic bags constitute 50% of plastic waste generated worldwide [2]. As conventional plastic materials are made of synthetic polymers that are naturally hard to degrade, plastic waste tends to accumulate in the environment. Recent studies have also demonstrated that over time plastic waste will be degraded into smaller fragments known as microplastics [3, 4]. Multiple studies have indicated the presence of microplastics in marine animals that are used for human consumptions, such as fish, seashells, and crabs [5, 6]. To date, there is no clear information on the impact of prolonged microplastic ingestion on health.

Biodegradable plastics are perceived as a solution to plastic waste accumulation issues. They refer to polymers that can be mineralized by microorganisms into water, carbon dioxide, methane, and biomass. Bioplastics, a subset of biodegradable plastics, are made of renewable natural polymers from plants such as cassava, corn, and potato starch [7] or from bacteria such as poly(3-hydroxyalkanoates) [8]. It should be noted that not all bioplastics are biodegradable, and likewise, not all biodegradable plastics are made of renewable natural resources [9, 10]. Another type of biodegradable plastic is made by incorporating metallic salt additives to petroleum-based plastics, such as low-density polyethylene (LDPE), more commonly known as oxo-biodegradable plastics [11]. Metallic salts act as pro-oxidants to initiate photo- or thermo- oxidation of the long-chained polyethylene polymer and break it down into lower molecular weight fragments that are presumably more susceptible to microbial attack [11, 12]. The biodegradability of oxo-biodegradable plastics, however, is subject to much debate as there are conflicting results on whether they are truly decomposed into simpler molecules (oxo-degradable) or merely broken down into microplastic fragments [10, 13]. Despite its controversial nature and the EU Parliament ban on oxo-degradable plastics, oxo-LDPE is one of the major types of plastic claimed as biodegradable and environmentally friendly circulating in the Indonesian market.

The physicochemical structure of the materials, the ambient circumstances, and the microbial populations engaged in biodegradation all affect how quickly biodegradable plastics degrade [9]. Abiotic and biotic factors may differ from one environment to another and overall influence the biodegradability of biodegradable plastics [14, 15]. Abiotic factors, including temperature, acidity, oxygen, humidity, and ultraviolet exposure, play a role in initiating abiotic hydrolysis in the main degradation step [15, 16]. The presence of oxygen determines biological reactions and the type of decomposers, including bacteria, fungi, archaea, or algae, that will further degrade plastic materials [17–20]. In oxygenic conditions, aerobic organisms utilize polymers as carbon and energy sources to generate carbon dioxide, while in limiting oxygen concentrations, anaerobic organisms decompose polymers and generate methane [21]. Most work on the microbial decomposition of biodegradable plastic decomposition is based on single pure cultures [18, 22–24], while microorganisms in the environment are more likely to occur as a consortium.

The impact of biodegradable plastic polymers used as agricultural plastic mulch film on soil microbiome has been assessed previously. Muroi et al. used polymerase chain reaction-denaturing gradient gel electrophoresis (PCR-DGGE) to analyze soil microbiome following poly (butylene adipate-co-terephthalate) (PBAT) film burial at a lab-controlled temperature of 30 °C over 7 months [25]. They showed that even though PBAT exposure led to the enrichment of Ascomycota, it did not significantly affect the bacterial composition in soil [25]. It should be noted that the breadth of information captured through PCR-DGGE is highly dependent on the scope of universal primers used in the study, as some primers may be biased towards Proteobacteria [26]. High-throughput sequencing analysis on the 16S rRNA gene further indicated that exposure of PBAT to soil over a shorter period time of 8 weeks at 15 °C increased the Proteobacteria and Actinobacteria relative abundance in Alpine and Arctic soil [27]. Poly (butylene succinate-co-adipate) (PBSA) film had a 28–33% molar mass reduction after 11 months of burial in soil under ambient climate and lab-controlled conditions, and this is accompanied by the enrichment of the aquatic fungi *Tetracladium* spp. and nitrogen-fixing bacteria [28]. In contrast, it is less well-known how the soil microbiome is affected by biodegradable plastic materials typically used for consumer products, such as carrier plastic bags.

This study monitors soil bacterial composition during the burial of two types of plastics marketed as biodegradable in Indonesia: cassava starch-based bioplastics and oxo-LDPE. We do not specifically attempt to quantify biodegradation according to any specific standards, but we rather assess how the bacterial population in soil may or may not have changed upon exposure to plastic products claimed as biodegradable. By identifying bacterial groups that arise in population over time, we will obtain preliminary insight into which bacteria may be able to utilize the biodegradable plastic material for their growth. Such information is important for identifying bacterial groups that may be used in the management of biodegradable plastic waste.

## Methods

### Sample preparation

Two types of carrier plastic bags marketed as biodegradable, i.e. the cassava starch-based bioplastic and oxo-LDPE, were purchased from e-commerce stores in Indonesia. Each type of plastic bag was procured from a separate store. These plastics were chosen based on their availability as consumer products in the Indonesian market. They were cut into random-sized sheets, weighed to 75 g batches, UV sterilized and buried at 10 cm depth in compost soil (Sahabat Tani brand; contains a mixture of guano, humus, manure, roasted rice husks, dolomite and cocopeat as described in the packaging) in separate plastic pots (30-cm diameter). The pots were incubated on an outdoor balcony covered from direct rainwater exposure for 30, 60, 90 and 120 days. One pot was assigned for each type of biodegradable plastic and incubation time. Soil with no plastic stored in the same conditions and collected from the same depth at each time point served as controls. The inner temperature and pH of soil were measured using a digital thermometer and a pH meter at each time point, respectively. At the end of each treatment, remaining plastic was weighed. For each treatment and time point, soil attached to the remaining plastic was pooled into a sterile centrifuge tube and stored at 4 °C prior to further analysis.

Both plastic bags were buried on the 9th February 2021. One pot for each plastic type and incubation time was subsequently sampled on four dates: 11th March 2021 (30 days), 10th April 2021 (60 days), 10th May 2021 (90 days), and 9th June 2021 (120 days). Over this period, the average daily temperatures ranged from 24.3–29.2 °C and the relative humidity was 74–93% [29].

### Soil DNA extraction

Total DNA from control soil and soil surrounding the remaining plastic was extracted using the Presto™ Soil DNA Extraction Kit (GeneAid, Taiwan) according to its manufacturer’s protocol and stored at − 20 °C. Soil samples that were not directly analyzed were stored at 4 °C for maximum 14 days. Total DNAs were visualized on a 1% agarose gel and quantified using NanoDrop™ 2000 (Thermo Fischer Scientific, USA).

### V3-V4 library preparation and high-throughput sequencing

The V3-V4 region of 16S rDNA was amplified using the primer pair 341f (5′–GTGCCAGCMGCCGCGGTAA-3′) and 806r (5′-CCGTCAATTCCTTTGAGTTT-3′) [30]. Amplicons were generated and tagged with unique sequence barcode for each sample with NEBNext® UltraTM DNA Library Prep Kit for Illumina. Sequencing was done using the Illumina platform at NovogeneAIT, Singapore.

### Bioinformatics analysis

Overlapping DNA fragments were merged using FLASH (V1.2.7) [31]. Quality filtering at Phred value ≥ Q20 [32] was done to achieve high quality clean tags according to QIIME (V1.7.0) [33]. Chimera sequences were removed by comparing the tags to SILVA database (http://www.arb-silva.de/) using UCHIME algorithm [34, 35]. Uparse software (V7.0.1090) was used to assign the sequences that have ≥97% similarity into the same operational taxonomic unit (OTU) [36]. Shannon and Simpson indices were calculated using QIIME (Version 1.7.0) and displayed with R software (Version 2.15.3). Representatives of each OTUs were screened with QIIME (Version 1.7.0) [37] using Mothur against SSUrRNA database of SILVA Database for species annotation at each taxonomic rank [38, 39]. The taxonomic relationship of all representative sequences was analyzed using MUSCLE (Version 3.8.31) [40].

## Results and discussion

### Plastic weight decrease

**Fig. 1:**
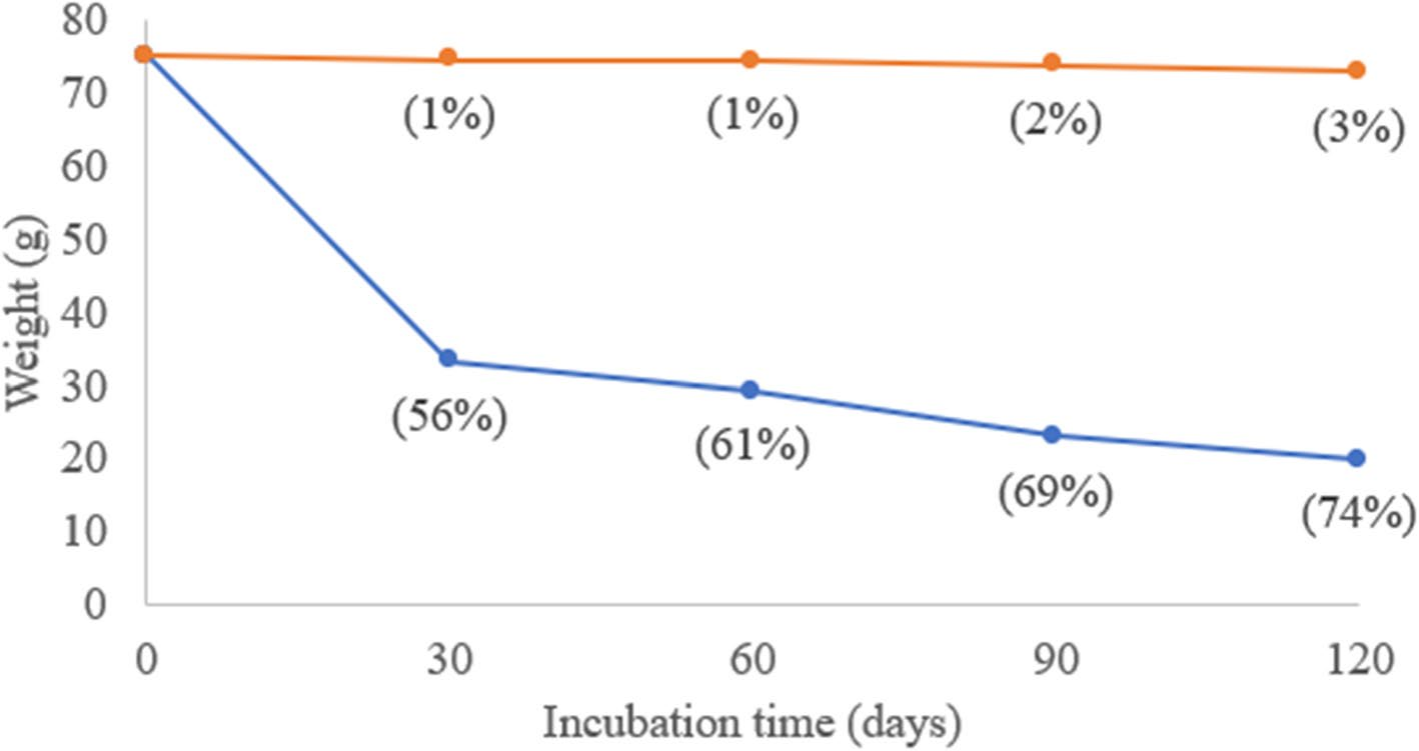
Starch-based bioplastic (blue) and oxo-LDPE (orange) weight reduction following their burial in compost soil over 120 days. Numbers in bracket represent weight decrease in percentage. Data shown were based on one experiment

After 30 days, the cassava starch-based bioplastic weight was reduced by 56%. The weight reduction was less drastic afterward, with a 61, 69, and 74% weight decrease by day 60, 90, and 120, respectively (Fig. 1). A study done by Accinelli et al. [41], in which a corn starch-based bioplastic bags was buried at 25 °C in the dark, also reported a 43% decrease in plastic weight after 90 days of incubation in compost soil. The starch component in this type of bioplastic serves as a carbon source for soil bacteria [42]. In contrast, oxo-LDPE weight was only reduced by 3% even after 120 days. This is in agreement with a previous study, which demonstrated only 1–1.5% degradation after 120 days burial in soil, even in combination with photooxidation pre-treatment. It should also be noted that the plastics samples in this study were weighed prior to UV sterilization and burial. UV light exposure induced the oxidative cleavage of the polyethylene chain in oxo-LDPE to produce lower molecular weight monomers or oligomers [43], and thus this treatment may also contribute to the 3% weight decrease. Further evaluations on biodegradation such as through surface and tensile strength analysis of the remaining plastics and carbon evolution study, which were not done in our experiment, would give more insight to which extent the plastics are being biodegraded. Soil pH (8.6 ± 0.2) and temperature (25 °C) in this study remained constant at all time points, meaning that the introduction of neither starch-based bioplastic nor oxo-LDPE affected both environmental parameters. Overall, our results confirm that not all types of plastics that are claimed as “biodegradable” gets degraded at the same rate in soil.

### Diversity indices

Sequencing of control group yielded 128,703 ± 17,381 (mean ± standard deviation) reads that were clustered into 2494 ± 246 OTUs. Sequencing of starch-based bioplastic treatment group yielded 115,495 ± 6073 reads that were clustered into 2464 ± 97 OTUs. Meanwhile, sequencing of oxo-LDPE treatment group generated 114,373 ± 4491 reads that were clustered into 2538 ± 196 OTUs.

Numbers following each sample code indicate the length of plastic burial in days. The value of Shannon index represents bacterial diversity, in which higher numbers indicate higher diversity. The Simpson index has a maximum value of 1, which signifies that all members of the population are present in equal abundance.

The diversity and richness of bacterial OTUs were quantified and expressed as Shannon and Simpson indices, respectively [44]. While the Shannon index over time fluctuated from 7.263 (day 0), 7.386 (day 30), 8.014 (day 60), 7.749 (day 90), to 7.537 (day 120) in soil with no plastic treatment, the bacterial diversity within soil introduced to both starch-based bioplastic and oxo-LDPE was directly proportional to the incubation time, in which the lowest diversity index was observed on day 30 (7.503 for Bt30 and 7.407 for Ot30) and the highest on day 120 (8.055 for Bt 120 and 8.591 for Ot120) (Table 1). Compared to the control group, soil treated with both types of plastic showed higher diversity over time, except on day 60. The Simpson index in soil treated with both types of plastic also showed that their presence led to a slight increase in bacterial richness over time, from 0.969 to 0.984 in starch-based bioplastic treatment and 0.971 to 0.990 in oxo-LDPE treatment.

**Table 1:** Diversity and richness of bacteria in soil following starch-based bioplastic and oxo-LDPE burial

Sample name	Treatment	Shannon index	Simpson index
Ct0	No plastic exposure (control)	7.263	0.952
Ct30		7.386	0.970
Ct60		8.014	0.985
Ct90		7.749	0.982
Ct120		7.537	0.972
Bt30	Starch-based bioplastic	7.503	0.969
Bt60		7.801	0.977
Bt90		7.908	0.982
Bt120		8.055	0.984
Ot30	oxo-LDPE	7.407	0.971
Ot60		7.589	0.972
Ot90		8.232	0.987
Ot120		8.591	0.990

### Bacterial community in compost soil introduced to cassava starch-based bioplastic

**Fig. 2:**
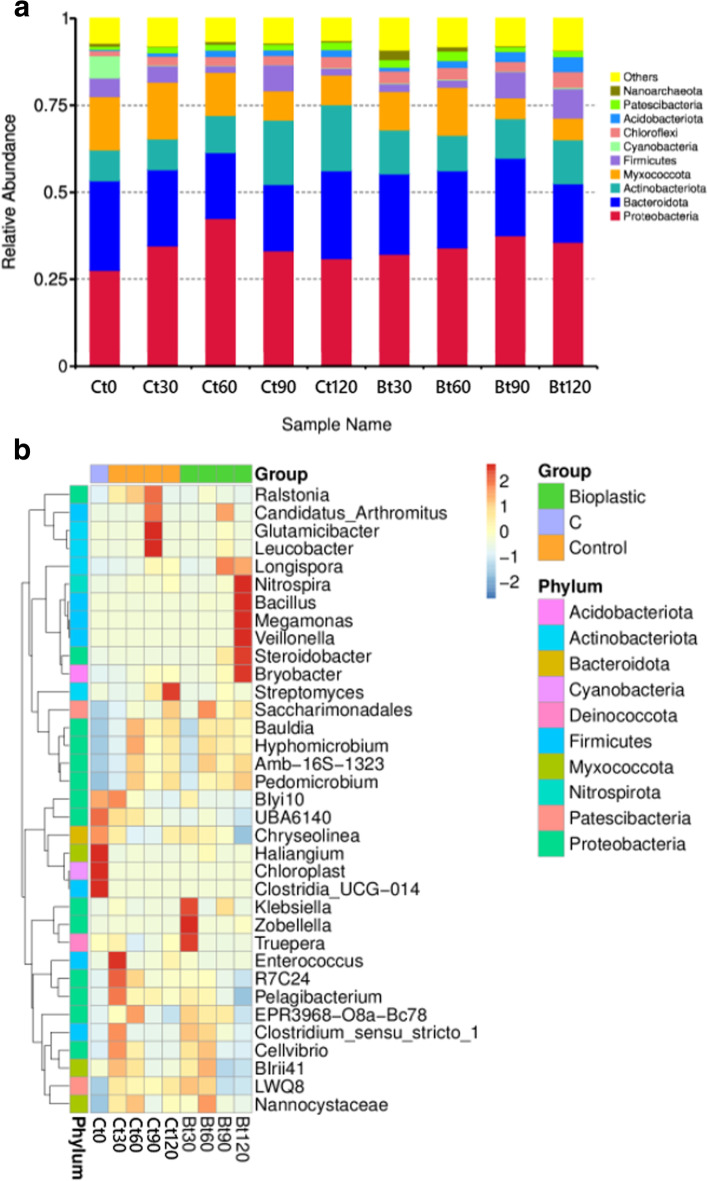
Abundance of bacterial phyla (**a**) and genus (**b**) in soil following burial of starch-based bioplastic (Bt) compared to control soil with no plastic addition (Ct). Numbers following sample codes indicate the length of plastic burial in days. Each bacterial phylum is represented by a specific color

The most dominant phyla in control soil and soil introduced with starch-based bioplastic were Proteobacteria, Bacteriodota, Actinobacteria, and Myxococcota (Fig. 2 a). This is in line with a report by Meng et al. that explored microbial succession during cow manure and corn straw composting [45]. At the beginning of the experiment, the control soil is composed mainly of Proteobacteria (27.6%), Bacteriodota (25.7%), Actinobacteria (8.9%), Myxococcota (15.3%). Minor phyla were also identified, including Firmicutes (5.4%), Cyanobacteria (6.4%), Chloroflexi (1.4%), Patescibacteria (0.9%), Acidobacteria (0.4%), and Nanoarchaeota (0.8%) (Fig. 2 a).

The above phyla remain present in compost soil with or without plastic exposure over time, but their composition changed (Fig. 2 a). Proteobacteria abundance was generally increased and by day 120, the population was slightly higher in soil exposed to starch-based bioplastic (35.7%) than in control soil (31.0%). Firmicutes relative abundance fluctuated in the control soil (5.4 → 4.7 → 1.9 → 7.4 → 1.9% at each time point), while soil treated with starch-based bioplastic the phyla showed an initial decrease at day 30 (2.3%) to 60 (2.1%), followed by further increase at day 90 (7.6%) and 120 (8.5%). A slight increase was observed for Actinobacteriota in both the control and treated soil, but by day 90 and 120, the abundance in control soil (18.5 and 19.1%, respectively) was relatively higher than in soil exposed to starch-based bioplastic-treated soil (11.4 and 12.7%, respectively). Acidobacteria abundance also increased over time in both soil, but the final abundance at day 120 was higher in starch-based bioplastic soil (4.3%) compared to the control (2.0%). In contrast, the Myxococcota and Nanoarchaeota population showed a constant decrease throughout burial in all samples. The relative abundance of Cyanobacteria also decreased drastically from 6.4% to 0.1–0.2% in all samples. Such a decrease is expected considering that there was limited sunlight exposure during plastic burial, a factor that is required by most members of this group of photosynthetic bacteria to grow [46].

Prevalent bacterial genera varied across all time points and some of these genera are known for their ability to degrade starch (Fig. 2 b). Starch is a polymeric carbohydrate that consists of repeated glucose molecules chained with glycosidic bonds. Amylase cleaves the glycosidic bonds in starch to generate oligo-, di-, or monosaccharides, all of which play a role as growth substrates for microorganisms [47]. Various species belonging to *Klebsiella,* which was detected in abundance on day 30, are known to produce enzymes for starch degradation, such as α-cyclodextrin glycosyltransferase, α-amylase, and pullulanase [48]. *Longispora* was abundant on day 90 and 120. *Longispora fulfa* tested positive in the starch hydrolysis assay [49]. Meanwhile, *Bacillus* and *Nitrospira* were abundant on day 120*.* Many *Bacillus* species are known to hydrolyze starch through the activity of α-amylase, an enzyme that cleaves the α-1,4 glycosidic bonds in starch [50, 51]*.* A study on functional genes among the tropical peat swamp bacterial community by Kanokratana et al. [38] indicated the prevalence of amylolytic genes belonging to *Nitrospira*, which indicates that members of this group play a role in starch degradation in the environment. The fact that none of the above bacterial groups consistently rose up in abundance across all time points indicates that starch-based bioplastic catabolism may occur through dynamic metabolic activities of various bacteria rather than relying on specific bacterial genera or species.

In contrast, there is a lack of evidence for starch degradation for some other bacterial genera that increased in abundance following the introduction of starch-based bioplastic. Past studies showed that members of *Zobellela* and *Truepera* (day 30) play a role in the decomposition of high-carbohydrate organic materials. Maity et al. [39] demonstrated the use of *Zobellela tiwanensis* strain DD5 to produce polyhydroxybutyrate using the starch-rich banana peels as a substrate. Meanwhile, *Truepera* was reported to thrive in compost enrichment samples [52, 53].

On day 60, there was an increase of Saccharimonadales abundance. To our knowledge, aside from another study that showed the enrichment of this bacterial order with polylactic acid exposure in soil [27], there is no other report that associates Saccharimonadales with biodegradable plastics in the environment. Saccharimonadales belongs to the phylum Saccharibacteria, which is a member of the superphylum Patescibacteria. Very little is known about their ecology and how they may play a role in starch degradation. Functional genome analysis showed that some members of Saccharibacteria were missing genes for de novo biosynthesis of essential amino acids, nucleotides, fatty acids, and cofactors [54], which indicated that they might require co-metabolism with other bacteria to survive in the environment. A member of Saccharibacteria isolated from wild oats rhizosphere showed that it feeds of plant exudates and its genome also indicates the prevalence of starch/glycogen and trehalose breakdown gene for D-glucose production [55]. More research needs to be done to understand their role in starch-based bioplastic degradation.

Aside from *Longispora*, *Bacillus* and *Nitrospira*, all other genera that increased in abundance on day 120, including *Megamonas, Steroidobacter, Veillonella*, and *Bryobacter,* have not been associated with starch hydrolysis thus far. There is, however, an indication that they made indirect contribution to starch degradation and/or organic material decomposition in general. *Megamonas* is mainly found in human faeces and the human gut microbiome. In an in vitro pea starch digestion model, Cui et al. [44] reported that *Megamonas* was found in a large number after 8 hours of digestion. *Veillonella* thrives in the gut of gnotobiotic rats fed with amylomaize starch by utilizing starch degradation products derived by the amylolytic bacteria *Eubacterium* [56]. *V. atypica* was also reported to co-exist and communicate with *Streptococcus godonii* during the early formation of dental plaque biofilm [57]. This study showed that the presence of *V. atypica* increased the expression of the amylase-encoding gene *amyB* in *S. godonii. Bryobacter aggregatus* gen. Nov., sp. nov., was reported to grow on starch, glucose, and maltose medium [58]. While there is no report on amylase activity in *Bryobacter* [58], it is possible that *Bryobacter* can use the starch degradation products such as maltose and glucose produced by starch degrading bacteria. Overall, this suggests that even though the above bacterial genera might not be involved in starch-based bioplastic degradation directly, they might thrive in soil by utilizing starch degradation intermediates or through other interactions within the bacterial community.

### Bacterial community in compost soil introduced to oxo-LDPE

**Fig. 3:**
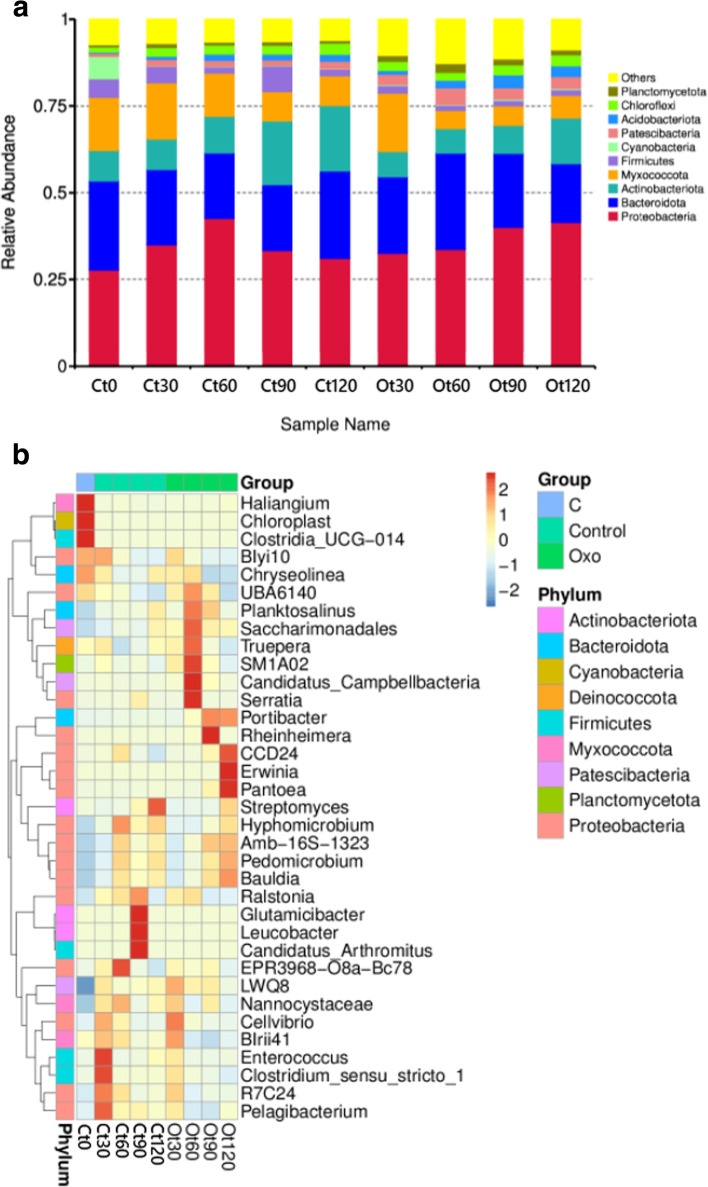
Abundance of bacterial phyla (**a**) and genus (**b**) in soil following burial of oxo-LDPE (Ot) compared to control soil with no plastic addition (Ct). Numbers following sample codes indicate the length of plastic burial in days. Each bacterial phylum is represented by a specific color

Similar with the observation for starch-based bioplastic treatment, the dominant phyla in control soil and soil introduced with oxo-LDPE were Bacteroidota, Proteobacteria, Actinobacteria, and Myxococcota (Fig. 3a). The relative abundance of Proteobacteria in control soil initially increased on day 30 (35.0%) and day 60 (42.6%) but decreased on day 90 (33.4%) ad day 120 (31.1%). Meanwhile, the Proteobacteria population in soil treated with oxo-LDPE increased steadily over time to 41.4% on day 120. As for Actinobacteria, an increase was observed in all samples from the initial 8.8% abundance at the beginning of the experiment to 18.9 and 13.2% on day 120 for control soil and oxo-LDPE-treated soil, respectively. Bacteriodota population fluctuated over time. Less dominant phyla were also present in both soil and showed slightly higher in abundance following oxo-LDPE introduction, including Patescibacteria (from the initial 0.9% abundance at the beginning of the experiment to 2.1 and 3.5% on day 120 for control soil and oxo-LDPE-treated soil, respectively) and Acidobacteriota (from the initial 0.4% abundance at the beginning of the experiment to 2.0 and 3.0% on day 120 for control soil and oxo-LDPE-treated soil, respectively). In addition, a small fraction of Planctomycetota was included in top 10 bacterial phylum detected in all soil samples. Whereas this phylum was stagnant in control soil, its relative abundance was increased on day 30 (1.7%) dan day 60 (2.6%), followed by further decrease on day 90 (1.7%) and day 120 (1.4%). Meanwhile, across all samples a decrease was observed for Myxococcota (from the initial 15.3% abundance at the beginning of the experiment to 8.6 and 6.5% on day 120 for control soil and oxo-LDPE-treated soil, respectively) and Firmicutes (from the initial 5.4% abundance at the beginning of the experiment to 1.9 and 1.6% on day 120 for control soil and oxo-LDPE-treated soil, respectively).

Only one genus associated with the degradation of the polyethylene backbone of oxo-LDPE emerged following its introduction in soil. *Serratia,* which was abundant on day 60 (Fig. 3 b), was reported to degrade polyethylene [59, 60]. Faster degradation of polyethylene was achieved when the cell-free supernatant of *Serratia marcescens,* compared to the viable bacterial cells, was applied to the plastic film [59]. This showed that the bacterium produced extracellular enzyme(s) to degrade polyethylene. In addition, several genera that have been detected in polyethylene- or microplastic-rich environment also arose at various time points, despite the lack of information on their direct contribution to polyethylene degradation. *Saccharimonadales* (day 60) was detected in microplastic-contaminated soil [61]. Similarly, *Rheinheimera* (day 90) was reported in abundance in microplastic-infested water [62]. *Pantoea* (day 120) was found in the gut of the polyethylene-degrading *Galleria mellonella* and *Tenebrio molitor* larvae that were kept on polyethylene-rich diet [63, 64]. *Portibacter* (day 90 and 120) was identified among major bacterial colonizers of polyethylene plastic debris [65]. Interestingly, as observed for starch-based bioplastic, no bacterium thrived continuously across all time points in soil introduced to oxo-LDPE. This indicate that both starch-based bioplastic and oxo-LDPE degradation may require a multitude of bacteria that will continue to shift over time.

Furthermore, the abundance of *Streptomyces*, members of which are known as polyethylene degraders [66, 67], was lower in soil exposed to oxo-LDPE than in control soil, albeit that their abundance continued to increase over time in both treatments. To initiate degradation, bacteria first need to colonize the surface of plastic by forming a biofilm layer known as “plastisphere” [68, 69]. The fact that the *Streptomyces* abundance was increasing over time in soil indicates that the bacteria, and perhaps other bacterial groups, may require longer than 120 days to establish succession on the surface of plastic and catabolize the material.

In soil exposed to either starch-based bioplastic or oxo-LDPE, we observed the elevated relative abundance of bacterial genera involved in nitrogen cycling. Starch-based bioplastic burial led to the increase of the nitrogen fixer *Longispora, Bacillus, Klebsiella,* and *Bryobacter* [70–73]. In the presence of this bioplastic, the population of other genera that play other roles in nitrogen cycling, such as the nitrification bacteria *Nitrospira* [74] and the denitrification bacteria *Steroidobacter* [75] and *Truepera* [76]*,* was also shown. Furthermore, Saccharimonadales, *Rheinheimera*, *Pantoea*, and *Serratia*, all of which were increased following oxo-LDPE burial in soil, were reported for their nitrogen fixating and plant growth promoting activities [77–80]. Correspondingly, a previous study showed that nitrogen-fixing bacteria promote the microbial breakdown of PBSA by enhancing the fungal abundance, accelerating the activity of enzymes that break down plastic, and influencing interactions with the plastic-fungal community [81]. This shed light on the complexity of interaction among different types of bacteria and other microorganisms within the soil microcosm, which overall determines the fate of biodegradable plastics in the environment.

This study is focused on soil bacterial community profiles during the introduction of commercial cassava starch-based bioplastic and oxo-LDPE. Fungal diversity analysis was not included as the availability of database and non-bias universal primers are lacking for this group of microorganisms at the time this study was conducted. In the future, prolonged incubation up to the point where oxo-LDPE has shown more signs or deterioration will provide a more in-depth view on microbial dynamics, particularly for the oxo-LDPE, which may require longer time to degrade.

## Conclusions

Cassava starch-based bioplastic was degraded faster in soil compared to oxo-LDPE. Our results indicated that the bacterial composition in soil changed over time with or without the introduction of either type of plastic. While the major bacterial phyla remained similar for all treatment in this study, the addition of both types of plastic led to a different shift in soil bacterial community. Various genera emerged at specific time points and none of them dominated the soil bacterial community continuously. They represent bacteria that might be directly involved in breaking down the plastic polymers, as well as those that survive by interacting with the degraders. Bacterial groups involved in nitrogen cycling also arose over time. Overall, this study suggests that the fate of biodegradable plastics in the environment is determined by a complex set of microbes that continues to change over time.

## Data Availability

The datasets generated and/or analysed during the current study are available in the NCBI repository, accession number PRJNA803316.
